# Subcutaneous sumatriptan: results of a peculiar, unpublished, comparative, double-blind, randomised, and controlled trial

**DOI:** 10.1007/s10194-011-0312-x

**Published:** 2011-03-01

**Authors:** Peer Tfelt-Hansen

**Affiliations:** Department of Neurology, Danish Headache Centre, University of Copenhagen, Glostrup Hospital, Glostrup, Denmark

To the Editor,

Aspirin plus metoclopramide, an antiemetic and prokinetic drug, for acute migraine treatment was introduced by Marcia Wilkinson at The City of London Migraine Clinic [1] and until the advent of sumatriptan, it was in several countries the standard treatment for migraine, see [2]. Sumatriptan, subcutaneous 6 mg and oral 100 mg, was introduced in 1991 [3]. Subcutaneous sumatriptan 6 mg resulted in headache relief (HR) (a decrease of headache from moderate or severe to none or mild) in 70–72% after 1 h [4, 5] and 86% after 2 h [4].Oral sumatriptan 100 mg (56% HR) was not superior to the oral combination of aspirin 1,000 mg and metoclopramide (ASP + M)10 mg (45%) in the first attack treated, but sumatriptan was better than the combination in the second and third attack [6].

From 1993 to 1994, a randomised, controlled trial (RCT) was performed in France. It had the title: “A randomised, multicentre, double-blind, double-dummy, parallel-group study to compare the efficacy and safety of subcutaneous sumatriptan with oral aspirin plus oral metoclopramide in the acute treatment of migraine” [7].

It was performed in 46 centres in France and showed, as expected, that subcutaneous sumatriptan 6 mg was superior (*p* < 0.001) to efferverscent aspirin 1,000 mg plus metoclopramide 10 mg from 30 to 120 min, see Table 1. Subcutaneous sumatriptan 6 mg was successful in 84% of patients at 2 h and similar results were reported in one pivotal RCT (86%) on subcutaneous sumatriptan [4]. The headache relief for ASP + M was 62% and somewhat higher than the 45% observed in a comparative RCT [6], see above, but in the range of the 56% headache relief observed in two other RCTs, with highly soluble lysine acetylsalicylate plus metoclopramide [8, 9]. The relative high-headache response for ASA + M could be due to the use of effervescent aspirin [7].

**Table 1:** Headache relief (HR) and therapeutic gain (percentage HR after subcutaneous sumatriptan 6 mg minus percentage HR after effervescent aspirin 1,000 mg plus metoclopramide) for the first-treated migraine attack

	Sumatriptan (*n* = 122)	ASP + M (*n* = 125)
Headache relief after 30 min		
Yes	62 (50%)	31 (24%)
No	62 (50%)	96 (76%)
*Therapeutic gain 26% (95% CI 14–37%)*		
Headache relief after 60 min		
Yes	87 (71%)	57 (45%)
No	35 (29%)	69 (55%)
*Therapeutic gain 26% (95% CI 14–38%)*		
Headache relief after 120 min		
Yes	99 (84%)	79 (62%)
No	19 (16%)	48 (38%)
*Therapeutic gain 22% (95% CI 11–32%)*		

The rationale for doing this RCT remains obscure. Why should an injection of an antimigraine drug (the optimal route of administration) be compared with an oral form of another antimigraine drug (a suboptimal form because of slower absorption)? The most likely reason is that this RCT was done solely for registration purposes in France. We don’t know whether it was done at the request of the French health authorities. Any way, this RCT, even if based only on the summary, was of good quality and the RCT remains unpublished 17 years later. Publication is an obligation of the clinical investigators [10] and this RCT should have been published many years ago.
